# The efficacy and safety of antimuscarinics for the prevention or treatment of catheter-related bladder discomfort: a systematic review and meta-analysis of randomized controlled trials

**DOI:** 10.1186/s13741-021-00217-0

**Published:** 2021-12-14

**Authors:** Zhongbao Zhou, Yuanshan Cui, Xiaoyi Zhang, Youyi Lu, Zhipeng Chen, Yong Zhang

**Affiliations:** 1 Department of Urology, Beijing TianTan Hospital, Capital Medical University, No.119 South 4th Ring West Road, Fengtai District, Beijing, 100070 China; 2 Department of Urology, The Affiliated Yantai Yuhuangding Hospital of Qingdao University, Yantai, 264000 Shandong China; 3 Department of Urology, PLA Rocket Force Characteristic Medical Center, No. 16 Xinwai Street, Xicheng District, Beijing, 100088 China; 4 Department of Urology, Weifang People’s Hospital, Weifang, 261000 Shandong China

**Keywords:** Antimuscarinics, Catheter related bladder discomfort, Randomized controlled trials, Prevention or treatment, Systematic review, Meta-analysis

## Abstract

**Objectives:**

This meta-analysis aimed to evaluate the efficacy and safety of antimuscarinics for the prevention or treatment of catheter related bladder discomfort (CRBD).

**Methods:**

The MEDLINE, EMBASE, and Cochrane Controlled Trials Register (from 1987 to July 2021) were used to search randomized controlled trials. The PRISMA checklists were followed. RevMan5.4.0 was used for statistical analysis.

**Results:**

Eleven studies involving 1165 patients were involved in the analysis. The study reported that the incidence of CRBD observed in the antimuscarinics group was significantly lower than that of the control group at 0-, 1-, 2-, and 6-h after drug therapy (*P* = 0.001, *P* < 0.0001, *P* = 0.0005, and *P* = 0.001, respectively). For side effects, there were not statistical differences between the antimuscarinics group and the control group, mainly including dry mouth (risk ratio (RR) = 1.31, 95% confidence interval (CI) = 0.95 to 1.80, *P* = 0.09), postoperative nausea and vomiting (RR = 1.02, 95% CI = 0.55 to 1.90, *P* = 0.87), facial flushing (RR = 1.06, 95% CI = 0.43 to 2.61, *P* = 0.90), and blurred vision (RR = 0.95, 95% CI = 0.35 to 2.58, *P* = 0.91). Besides, rescue analgesics were required less in the antimuscarinics group than in the control group (RR = 0.51, 95% CI = 0.32 to 0.80, *P* = 0.003).

**Conclusions:**

Compared with the control group, the antimuscarinics group had a significant improvement on CRBD, the patients were well tolerated and the use rate of rescue analgesics was low.

## Introduction

The temporary insertion of catheter to improve intraoperative micturition and evaluation of perioperative voiding volume is essential operation for patients undergoing surgery. However, the incidence of catheter-related bladder discomfort (CRBD) immediately after operation is as high as 47~90% (Bala et al. 2012; Kunin 2001).

Patients describe CRBD as discomfort in the suprapubic area caused by indwelling a catheter or a burning sensation with micturition impulse (Binhas et al. 2011). As one of the most painful postoperative complications, CRBD increases the incidence of postoperative pain and reduces the quality of perioperative recovery (Maro et al. 2014). The exact pathophysiology of CRBD is not clear. However, its symptoms are similar to overactive bladder (frequent and urgent micturition with or without urgent urinary incontinence) caused by the involuntary contraction of bladder mediated by muscarinic receptors located in the urothelium and efferent nerve (Abrams et al. 2014; Andersson 1999). Five muscarinic receptor subtypes (M1–M5) in vivo mediate distinct physiological functions according to their locations and receptor subtypes (Bai et al. 2015). All of them have been detected in the bladder; it was thought that the most physiologically relevant subtypes were M2 and M3, with M2 being more abundant in the detrusor muscle but M3 being more active in eliciting detrusor contraction (Burden and Abrams 2016). M2 and M3 are mainly located in the detrusor muscle, urothelium, and efferent nerves (Yamanishi et al. 2001). Catheterization can stimulate the afferent nerve of bladder, leading to the release of acetylcholine, which causes muscarinic receptor-mediated involuntary contractions of detrusor muscle (Yamanishi et al. 2001).

At present, antimuscarinics have been reported to be an effective drug in preventing and treating CRBD, including tolterodine, solifenacin, oxybutynin, butyl scopolamine, and so on. But sometimes, side effects were observed. A recent review indicated that antimuscarinics seemed to achieve a better improvement in the clinical symptoms and a significant reduction in the incidence of CRBD compared with placebo, although these studies observed a high incidence of intervention-related side effects, in general, patients tolerated these treatments well (Bai et al. 2015). However, there was no comprehensive meta-analysis to assess the efficacy and safety of antimuscarinics in CRBD.

Therefore, we did a meta-analysis to evaluate the characteristic of antimuscarinics for CRBD in patients who underwent various surgical procedures where a catheter was inserted.

## Materials and methods

### Study protocol

The Preferred Reporting Items for Systematic Reviews and Meta-Analyses (PRISMA) checklist was used to analysis of randomized controlled trials (RCTs) (Moher et al. 2010).

### Inclusion criteria

RCTs met the following criteria: (1) antimuscarinics in preventing or treating CRBD were studied; (2) the study should consist of analyzable data referred to efficacy and safety, mainly including the number of patients with CRBD, dry mouth, postoperative nausea and vomiting (PONV), facial flushing, blurred vision, or rescue analgesics; and (3) the full text of articles should be available. If the above criteria were not met, the study was removed from our study.

### Search strategy

We searched MEDLINE, Embase, and Cochrane Controlled Trials Register databases to identify RCTs published before July 2021 using the following key words: antimuscarinics, anticholinergic, solifenacin, darifenacin, tolterodine, oxybutynin, scopolamine, glycopyrrolate, atropine, CRBD, and RCT. We confined our search to published studies in English only. The author reviewed the references of articles as well. We also tried to contact the authors of articles that we could not view full text.

### Trial selection

Three authors independently identified relevant studies according to inclusion criteria. Any discrepancies were recorded, discussed, and settled in a negotiated manner. If the identical study was published in different journals or at different time, the latest study was included in the analysis. However, if a group of patients was involved in two or more studies, each study may be included.

### Quality assessment

We used the Cochrane Handbook for Systematic Reviews of Interventions 2nd Edition to assess the quality of each study (Cumpston et al. 2019). We evaluated the methodological quality according to selection bias, performance bias, detection bias, attrition bias, reporting bias, and other bias. The quality of each study was classified as one of three degrees: “+” if the study satisfied all quality criteria, the study had a low risk of bias; “?” if the study had one or more ambiguous quality criteria, the study had a moderate risk of bias; “−” if the study met few quality criteria, the study had a high risk of bias. All authors assessed the quality of RCTs and agreed with the final results.

### Data extraction

We collected the following data from each study: (1) the name of first-author and the publishing year of the article, (2) study design, (3) the method of therapy; (4) sample size, (5) catheter type, (6) timing of administration, (7) anesthesia type; (8) ASA score and (9) surgery type; (10) basic information of patients, including age, sex, weight, duration of surgery and anesthesia; and (11) number of loss to follow-up, whether to calculation of sample size, the method of statistical analysis, and whether to intention-to-treat (ITT) analysis.

### Statistical analysis

The present meta-analysis was carried out by using Review Manager version 5.4.0 (Cochrane Collaboration, Oxford, UK) (Cumpston et al. 2019). The results were expressed as the risk ratio (RR) for discontinuous outcomes with 95% confidence intervals (CI) (DerSimonian and Laird 1986). We assessed the degree of heterogeneity with Cochrane’s *Q* tests and *I*^2^ statistics. *P* value ≤ 0.05 or *I*^2^ ≥ 50% reflected a significant heterogeneity. To reduce the heterogeneity, a random-effects model was used in the study. *P* < 0.05 was considered statistically significant. Due to the insufficient number of included studies, we did not perform subgroup analysis to analyze the source of heterogeneity.

## Results

### Study selection process and characteristics of studies

**Fig. 1:**
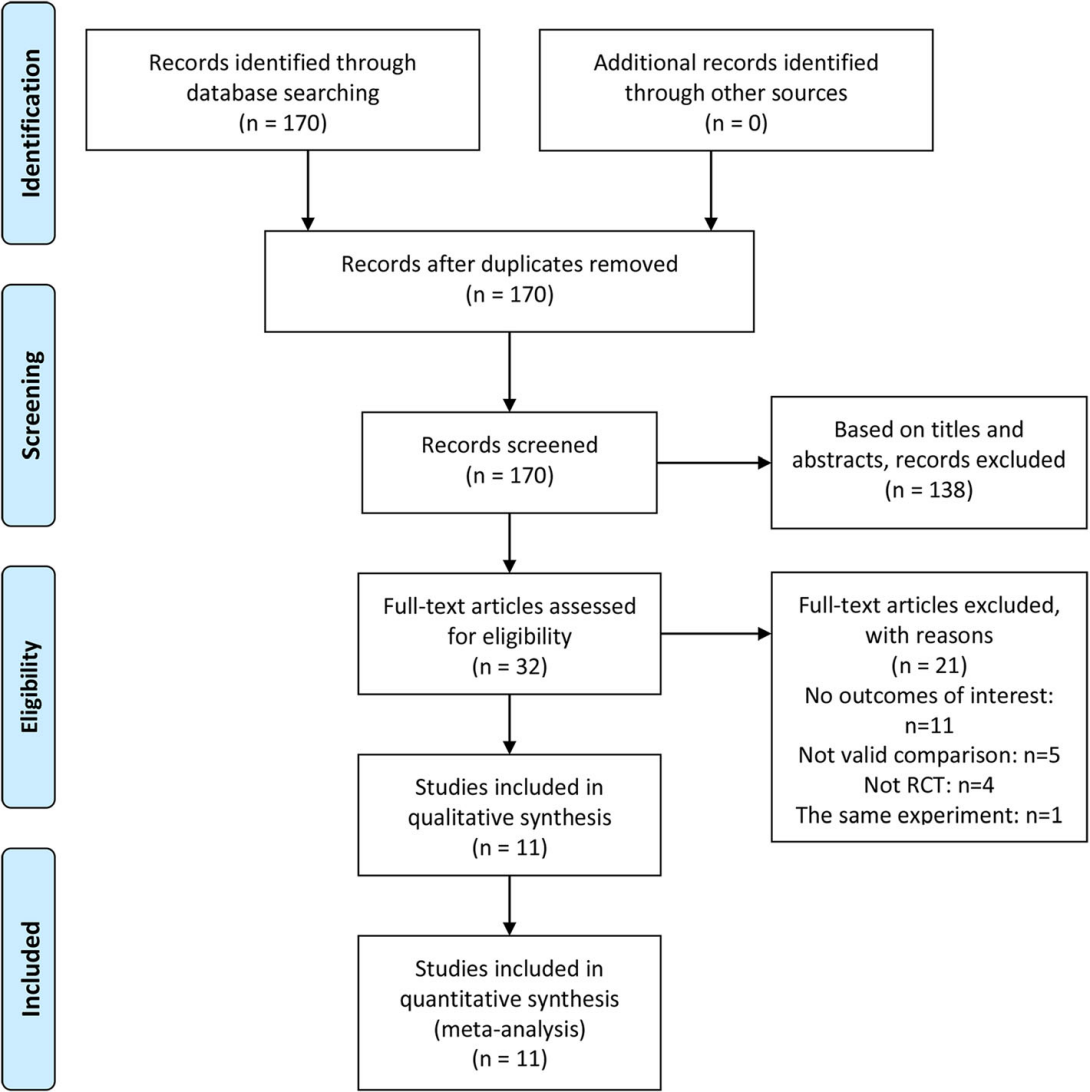
A flow diagram of the study selection process

Our search strategy found 170 articles in these databases. After reviewing their abstracts and titles, we ruled out 138 articles. Among the remaining 32 articles, 21 articles were excluded for lack of analyzable data. Finally, eleven studies (Agarwal et al. 2006; Agarwal et al. 2005; Chung et al. 2017; Kim et al. 2016; Maghsoudi et al. 2018; Nam et al. 2015; Ryu et al. 2013; Sabetian et al. 2017; Şahiner et al. 2020; Srivastava et al. 2016; Tauzin-Fin et al. 2007) involving 1165 patients were studied in the analysis (Fig. 1). The basic characteristics of RCTs were listed in Table 1 and Table 2.

**Table 1:** The details of individual study

Study	Study design	Therapy in experimental group	Therapy in control group	Sample size		Catheter type	Timing of administration	Anesthesia type	ASA score	Surgery type
				Experimental	Control					
Agarwal et al. 2005	RCT	Tolterodine 2 mg oral	Placebo	50	165	16 Foley	1 h before induction of anesthesia	General anesthesia	I/II	Urologic surgery for kidney and ureter
Agarwal et al. 2006	RCT	Oxybutynin 5mg / Tolterodine 2 mg oral	Placebo	78/78	78	16 Foley	1 h before induction of anesthesia	General anesthesia	I/II	Percutaneous nephrolithotomy surgery
Tauzin-Fin et al. 2007	RCT	Oxybutynin 5 mg sublingual	Placebo	23	23	16 Foley	Every 8 h for the first 24 h after surgery	General anesthesia	I/II	Radical retropubic prostatectomy
Ryu et al. 2013	RCT	Butyl scopolamine 20 mg iv	Saline	28	29	16/18 Foley	After reporting CRBD	General anesthesia	I/II	Urethrolithotomy, nephrolithotomy and radical retropubic prostatectomy
Nam et al. 2015	RCT	Butyl scopolamine 20 mg iv	None	49	50	14 Foley	Intravenously immediately before the end of the operation	General anesthesia	I/II	Stomach, hepatobiliary, colorectal operation
Chung et al. 2017	RCT	Solifenacin 5 mg oral	None	62	72	18 Foley	The day before, the day of, and the day after surgery	General anesthesia	NA	Transurethral resection of bladder tumor
Srivastava et al. 2016	RCT	Solifenacin 5 mg/Darifenacin 7.5 mg oral	Placebo	30/30	30	16 Foley	1 h prior to induction of anesthesia	General anesthesia	I/II	Elective spine surgery
Sabetian et al. 2017	RCT	Hyoscine *N*-butyl bromide 20 mg iv	Saline 1ml iv	24	26	22 Foley	Intravenously before the induction of anesthesia	General anesthesia	I/II	Transurethral resection of prostate
Maghsoudi et al. 2018	RCT	Tolterodine 2 mg oral	Vitamin C 250 mg oral	50	70	16 Foley	1 h before surgery	General anesthesia	I	Percutaneous nephrolithotomy
Kim et al. 2016	RCT	Glycopyrrolate 0.3 mg iv	Saline 1.5 ml iv	30	30	16 Foley	Intravenously before the induction of anesthesia	General anesthesia	I/II	Ureteroscopic removal of ureter stone
Şahiner et al. 2020	RCT	Atropine 15 μg/kg iv	Placebo	30	30	16-20 Foley	The end of the surgery	General anesthesia	I/II/III	Transurethral resection

*RCT* randomized controlled trial, *ASA* American Society of Anesthesiologists, *NA* not available, *CRBD* catheter related bladder discomfort

**Table 2:** The characteristics of patients

Study	Age (years) Mean ± SD (Range)		Sex (Male/Female)		Weight (kg) Mean ± SD		Duration of surgery (min) Mean ± SD		Duration of anesthesia (min) Mean ± SD		Loss to follow-up	Calculation of sample size	Statistical analysis	ITT analysis
	EXP	CON	EXP	CON	EXP	CON	EXP	CON	EXP	CON				
Agarwal et al. 2005	40.2 ± 13.6	42.6 ± 14.4	33/17	114/51	56.90 ± 8.09	58.24 ± 11.4	NA		NA		0	Yes	*T* tests; Chi-square test;	No
Agarwal et al. 2006	45.6 ± 13.2; 44.4 ± 12.8	43.6 ± 14.4	40/38; 39/39	42/36	55.4 ± 12.6; 56.9 ± 10.2	57.3 ± 11.4	NA		NA		0	Yes	Chi-square test; ANOVA	No
Tauzin-Fin et al. 2007	65.8 (54-72)	61.1 (54-75)	Male		74.1 ± 9.1	78.1 ± 13.8	188.2 ± 31.3	181.8 ± 33.8	NA		0	Yes	*T* tests; Chi-square test	No
Ryu et al. 2013	61 (22-70)	61 (24-69)	Male		67 ± 10	70 ± 11	178 ± 66	190 ± 76	224 ± 70	238 ± 82	0	Yes	*T* tests; Chi-square test	No
Nam et al. 2015	58 ± 10	60 ± 9	Male		69 ± 11	67 ± 9	154 ± 89	160 ± 95	198 ± 95	203 ± 101	0	Yes	Chi-square test; ANOVA	No
Chung et al. 2017	66 (43-84)	68 (26-85)	51/11	61/11	NA		NA		NA		0	Yes	ANOVA; *T* tests	No
Srivastava et al. 2016	46.8 ± 9.6; 43.0 ± 7.9	48.2 ± 10.2	24/6; 26/4	25/5	63.1 ± 8.6; 65.0 ± 10.3	61.1 ± 9.5	149.8 ± 28.5; 161.5 ± 40.5	156.7 ± 35.8	NA		0	Yes	ANOVA	No
Sabetian et al. 2017	64.95 ± 7.88	63.34 ± 9.22	Male		66.29 ± 6.77	65.61 ± 7.21	73.12 ± 20.42	76.34 ± 23.64	NA		0	Yes	*T* tests; Chi-square test; ANOVA	No
Maghsoudi et al. 2018	44.4 ± 9.7	44.1 ± 12.2	NA		25.9 ± 3.1 (BMI, kg/m^2^)	25.0 ± 4.2 (BMI, kg/m^2^)	97.9 ± 19.7	105.9 ± 23.4	NA		0	Yes	*T* tests; ANOVA	No
Kim et al. 2016	48.6 ± 12.2	50.2 ± 14.5	29/11	33/10	70.4 ± 12.8	70.2 ± 11.0	18.7 ± 12.2	23.0 ± 15.9	37.9 ± 13.1	39.3 ± 19.1	0	Yes	Chi-square test; Fisher’s exact test; *T* tests	No
Şahiner et al. 2020	59.7 ± 2.8	58.7 ± 2.5	25/5	25/5	27.9 ± 3.9 (BMI, kg/m^2^)	27.2 ± 3.4 (BMI, kg/m^2^)	41.5 ± 18.8	30.7 ± 14.6	53.3 ± 21.5	38.0 ± 17.8	0	Yes	Mann-Whitney U-test; T-tests; Chi-square test;	No

*EXP* experimental group, *CON* control group, *ITT* intention-to-treat, *ANOVA* analysis of variance, *SD* standard deviation, *NA* not available

### Quality of individual studies

**Fig. 2:**
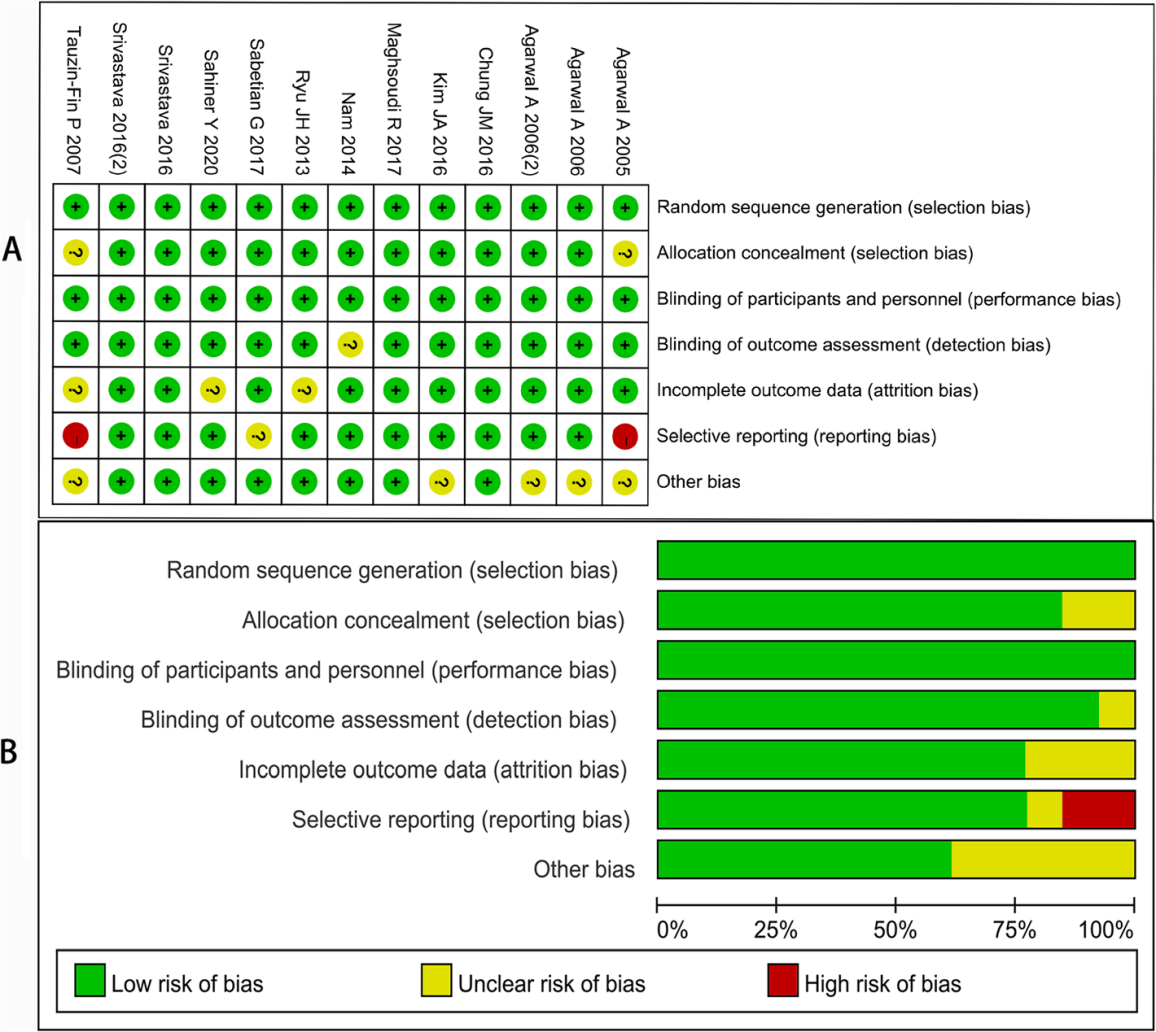
Risk of bias summary (**A**) and graph (**B**)

**Fig. 3:**
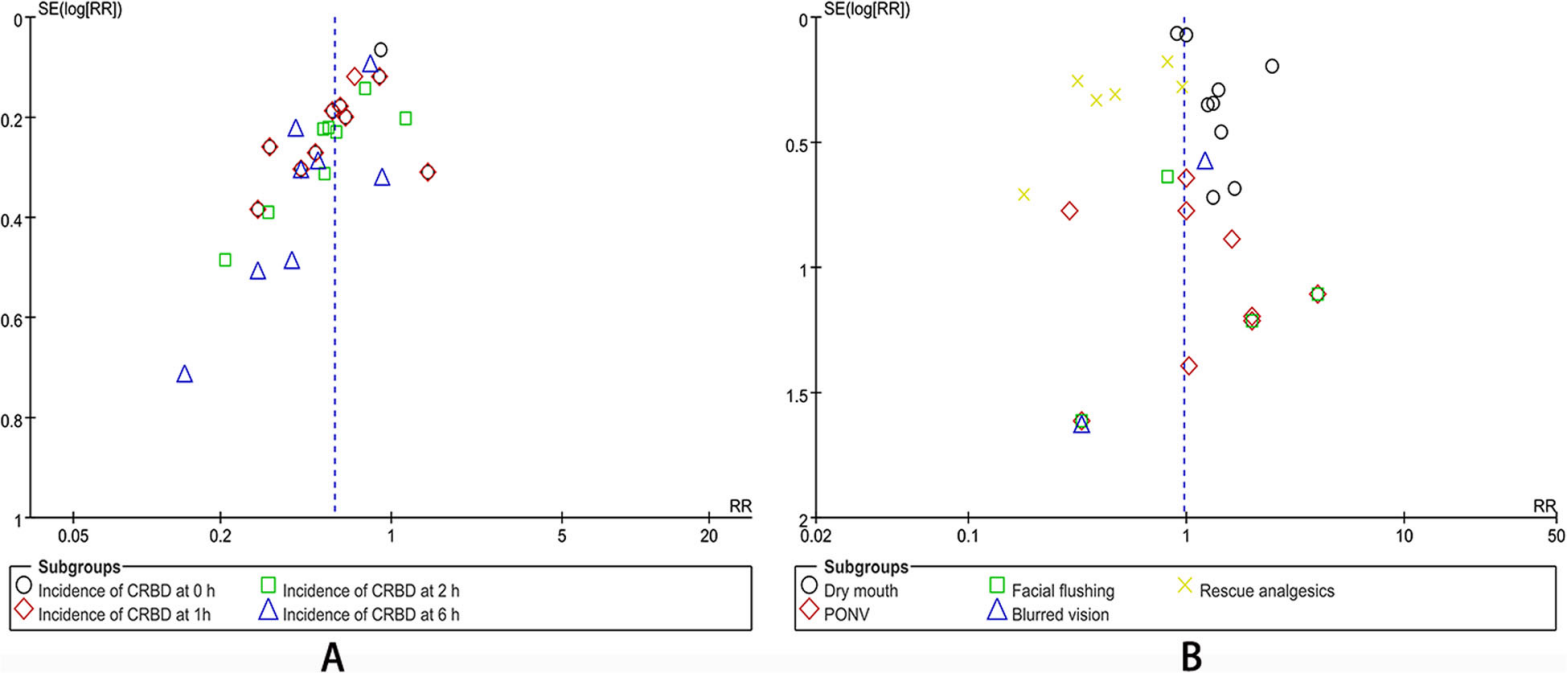
Funnel plot of the studies included in our meta-analysis. *RR* risk ratio, *PONV* postoperative nausea and vomiting

Eleven studies included in the analysis were RCTs. All studies had an appropriate number of participants to analyze, and no study showed intention-to-treat analysis (Table 2). Risk of bias summary and graph were showed in Fig. 2. The funnel plot showed a qualitative estimate of the publication bias of the study. The icons of each indicator were evenly distributed on both sides of the vertical line, which implied that no evidence of bias was found (Fig. 3).

### Antimuscarinics versus placebo on the incidence of CRBD

Eleven RCTs including 1059 patients (509 in the antimuscarinics group and 550 in the control group) were used to assess the impact of antimuscarinic on the incidence of CRBD.

#### The incidence of CRBD at 0 h

**Fig. 4:**
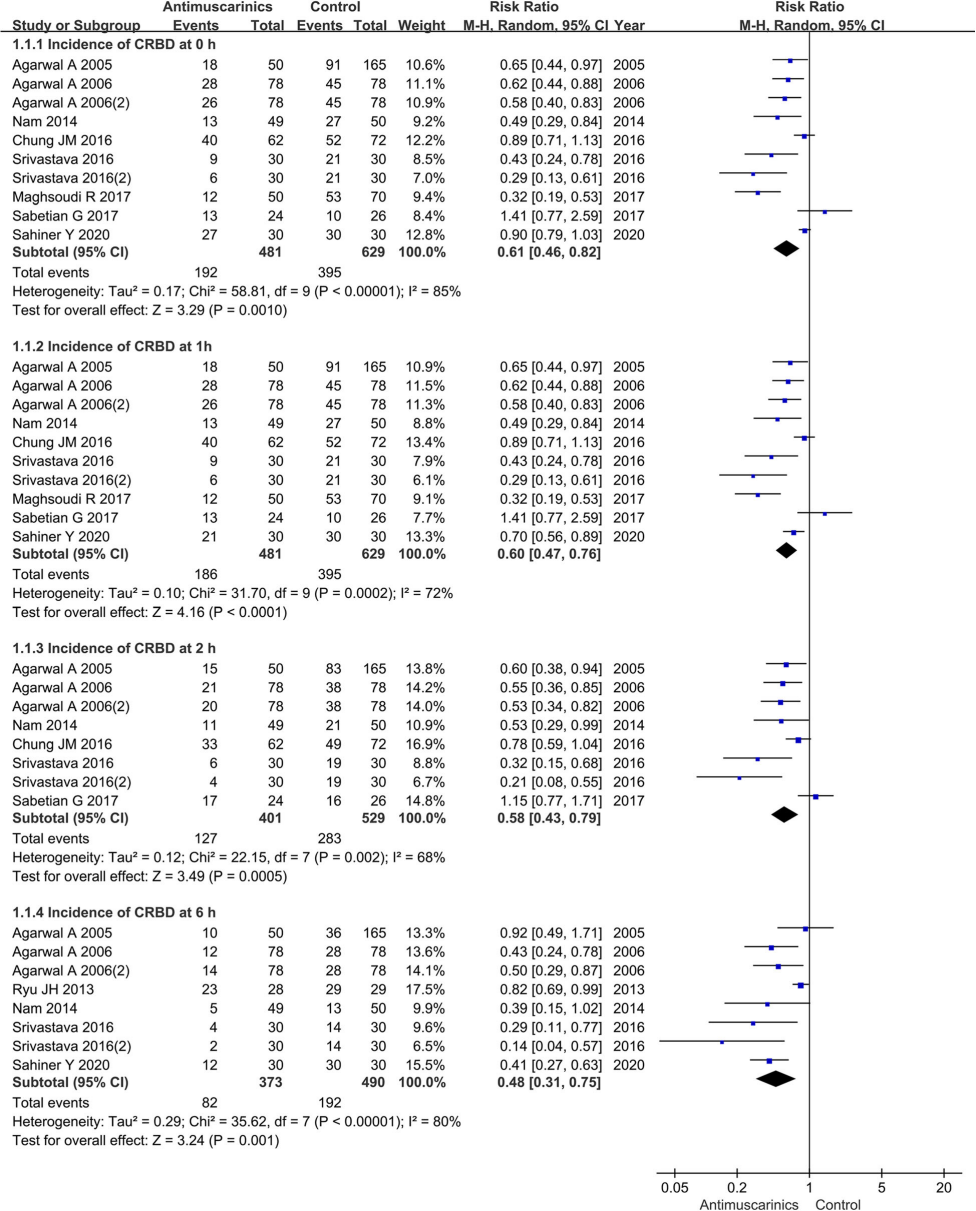
The incidence of CRBD in antimuscarinics versus placebo. *M*-*H* Mantel-Haenszel, *CI* confidence interval, *df* degrees of freedom, *RR* risk ratio

The analysis was supplied by ten RCTs including 1002 patients, which showed heterogeneity *P* value of < 0.00001 and *I*^2^ of 85%. Forest plots drew a RR of 0.61 and 95% CI of 0.46 to 0.82 (*P* = 0.001), which indicated that patients treated with antimuscarinics had a lower incidence of CRBD than those treated with placebo at 0-h postoperatively (Fig. 4).

#### The incidence of CRBD at 1 h

The analysis was supplied by ten RCTs including 1002 patients, which showed heterogeneity *P* value of 0.0002 and *I*^2^ of 72%. Forest plots drew a RR of 0.60 and 95% CI of 0.47 to 0.76 (*P* < 0.0001), which indicated that patients treated with antimuscarinics had a statistical reduction of CRBD than those treated with placebo at 1-h postoperatively (Fig. 4).

#### The incidence of CRBD at 2 h

The analysis was supplied by eight RCTs including 822 patients, which showed heterogeneity *P* value of 0.002 and *I*^2^ of 68%. Forest plots drew a RR of 0.58 and 95% CI of 0.43 to 0.79 (*P* = 0.0005), which indicated that patients treated with antimuscarinics had a statistical reduction of CRBD than those treated with placebo at 2-h postoperatively (Fig. 4).

#### The incidence of CRBD at 6 h

The analysis was supplied by eight RCTs including 755 patients, which showed heterogeneity *P* value of < 0.00001 and *I*^2^ of 80%. Forest plots drew a RR of 0.48 and 95% CI of 0.31 to 0.75 (*P* = 0.001), which indicated that patients treated with antimuscarinics had a statistical reduction of CRBD than those treated with placebo at 6-h postoperatively (Fig. 4).

### Antimuscarinics versus placebo on the incidence of adverse event

**Fig. 5:**
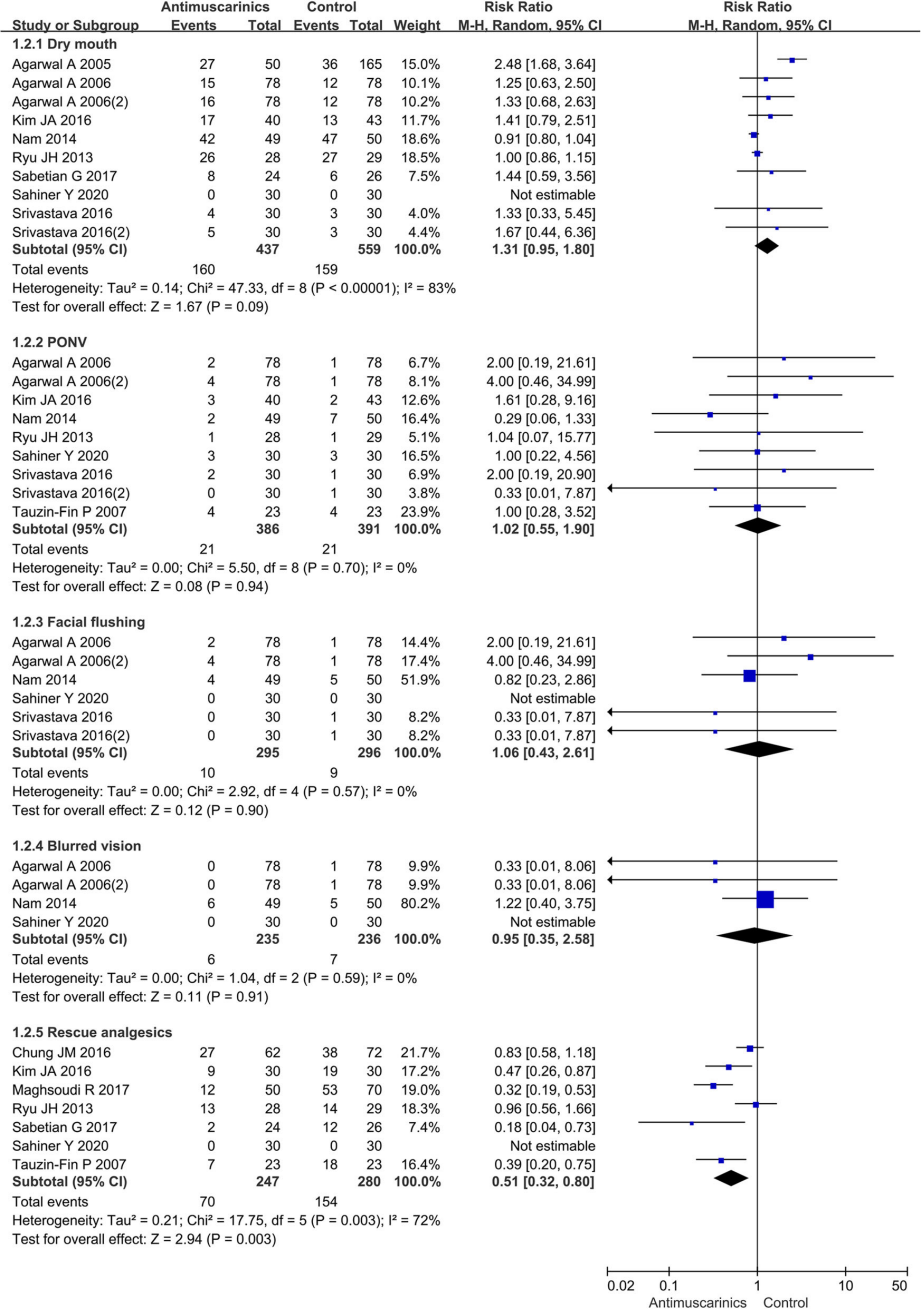
The incidence of adverse events in antimuscarinics versus placebo. *M*-*H* Mantel-Haenszel, *CI* confidence interval, *df* degrees of freedom, *RR* risk ratio

The adverse event including dry mouth, PONV, facial flushing, and blurred vision was reported in the included RCTs. The number of patients that needed rescue analgesics postoperatively in two groups was analyzed in our study. The results showed that there was no difference in the incidence of adverse effects between the antimuscarinics and control groups. Rescue analgesics were required less for patients in the antimuscarinics group than those in the control group (Fig. 5).

#### Dry mouth

Ten RCTs involving 888 patients reported the occurrence of dry mouth, which showed heterogeneity *P* value of < 0.00001 and *I*^2^ of 83%. The result indicated that antimuscarinics were not significantly different from placebo in the incidence of dry mouth (RR 1.31, 95% CI 0.95–1.80, *P* = 0.09).

#### PONV

Nine RCTs involving 669 patients reported the occurrence of PONV, which showed heterogeneity *P* value of 0.70 and *I*^2^ of 0%. The result showed that there was no statistical difference between the two groups in the incidence of PONV (RR 1.02, 95% CI 0.55–1.90, *P* = 0.94).

#### Facial flushing

Six RCTs involving 483 patients reported the occurrence of facial flushing, which showed heterogeneity *P* value of 0.57 and *I*^2^ of 0%. The result showed that there was no statistical difference between the two groups in the incidence of facial flushing (RR 1.06, 95% CI 0.43–2.61, *P* = 0.90).

#### Blurred vision

Four RCTs involving 393 patients reported the occurrence of blurred vision, which showed heterogeneity *P* value of 0.59 and *I*^2^ of 0%. The result showed that there was no statistical difference between the two groups in the incidence of blurred vision (RR 0.95, 95% CI 0.35–2.58, *P* = 0.91).

#### Rescue analgesics

Seven RCTs involving 527 patients reported the number of rescue analgesics, which showed heterogeneity *P* value of 0.003 and *I*^2^ of 72%. The result showed that the number of patients who needed rescue analgesics postoperatively was less in the antimuscarinics group compared with the control group (RR 0.51, 95% CI 0.32–0.80, *P* = 0.003).

## Discussion

Clinically, patients with indwelling urinary catheter were reported to suffer from a variety of catheter-related symptoms, such as suprapubic pain, frequency, urgency, and urge incontinence (Agarwal et al. 2005; Akça et al. 2016). Further, patients may have behavioral responses to CRBD, including agitation, loud complaints, and attempt to remove the bladder catheter accompanied by a burning sensation in the urethra, which resulted in the increased incidence of postoperative complications (Binhas et al. 2011).

Antimuscarinics have been widely used to treat overactive bladder for many years (White and Iglesia 2016). Because the symptoms of CRBD and overactive bladder (OAB) were similar, various muscarinic antagonists have been used in the treatment of CRBD. Antimuscarinics can block the muscarinic receptors of detrusor and prevent the stimulation of neurotransmitter acetylcholine. Thereby, they can reduce the frequency and intensity of detrusor contraction. Moreover, antimuscarinics have been proved to inhibit bladder-afferent mechanisms and increase the capacity of bladder (Dimitropoulos and Gravas 2015). Based on these pathways, antimuscarinics can effectively prevent or alleviate CRBD. In our study, it was also found that antimuscarinics statistically decreased the incidence of CRBD. However, antimuscarinics not only function in the bladder, but also in other parts of the body with muscarinic receptors which can result in some side effects, such as dry mouth, PONV, facial flushing, etc. (Abrams and Andersson 2007).

This meta-analysis identified that the incidence of CRBD had a statistically significant reduction at 0-, 1-, 2-, and 6-h after surgery for patient treated with antimuscarinics (*P* = 0.001, *P* < 0.0002, *P* = 0.0005, and *P* = 0.001, respectively). Safety assessment indicated that there were no statistical differences between antimuscarinics and placebo for side effects including dry mouth (*P* = 0.09), PONV (*P* = 0.94), facial flushing (*P* = 0.90), and blurred vision (*P* = 0.91). Besides, the number of patients who needed rescue analgesics postoperatively was less in the antimuscarinics group compared with the control group (*P* = 0.003). After a systematic analysis of the results, the study suggested the superiority of antimuscarinics in overcoming CRBD compared with placebo.

A better understanding of the pathophysiology of CRBD was the key to better management and reduction of incidence (Bai et al. 2015). Based on this concept, a variety of treatment options have been implemented, such as the insertion of catheter can cause detrusor contraction and the activity of inflammatory mediators, thereby promoting the synthesis of prostaglandins (PG) (Andersson 2010). Therefore, paracetamol, as a PG synthesis inhibitor, may improve the incidence and symptoms of CRBD (Ergenoglu et al. 2012). In addition, doctors should consider the occurrence of side effects when deciding which drug to use. For example, antimuscarinics (e.g., solifenacin and oxybutynin) are oral drugs that can cause adverse effects including dry mouth, facial flushing, and blurred vision. Therefore, it is necessary to evaluate its dose-response titration of each drug and the effect of treating CRBD (Bai et al. 2015). Simultaneously, it is noteworthy that preoperative periurethral local anesthesia infiltration can reduce immediate postoperative pain, but this method also needs to be further evaluated to determine its effect and safety (Kumar et al. 2013; Tommaselli et al. 2014).

The studies included in this analysis were all RCTs, which increased the strength of the results. Although all included studies were of high quality, our study also had some limitations. Firstly, this meta-analysis only included 11 studies with small sample size, which was limited to the number of relevant original studies. Secondly, the patient’s perioperative indicators may be inconsistent, including different sizes of catheter, different kinds of medications, different doses of medications, different timing of administration, and different ASA score. Thirdly, the patients included in the analysis have undergone various types of surgery. Among them, patients undergoing transurethral surgery had a higher incidence of CRBD, which may have an impact on the results. Finally, this paper involved the treatment or prevention of CRBD, but we cannot conduct a subgroup-analysis due to the small number of included articles, which may lead to different results. Therefore, high-quality RCTs were needed to explore the efficacy and safety of antimuscarinics for CRBD.

## Conclusions

Compared with the control group, the antimuscarinics group had a significant improvement on CRBD, the patients were well tolerated, and the use rate of rescue analgesics was low.

## Data Availability

All data generated or analyzed during this study are included in this article.

## Abbreviations

CRBD: Catheter related bladder discomfort
PRISMA: Preferred Reporting Items for Systematic Reviews and Meta-Analyses
RCTs: Randomized controlled trials
RR: Risk ratio
CI: Confidence interval
PONV: Postoperative nausea and vomiting
OAB: Overactive bladder
